# Carcinome papillaire intra-kystique du sein: à propos de trois cas

**DOI:** 10.11604/pamj.2014.18.207.4519

**Published:** 2014-07-07

**Authors:** Abderrahman El Mazghi, Touria Bouhafa, Kaoutar Loukili, Hanan El Kacemi, Issam Lalya, Taieb Kebdani, Khalid Hassouni

**Affiliations:** 1 Service de Radiothérapie, CHU Hassan II, Fès, Maroc; 2 Service de Radiothérapie, Institut National d'Oncologie, Rabat, Maroc; 3 Service de Radiothérapie, HIM Mohamed V, Rabat, Maroc

**Keywords:** Carcinome, papillaire, intra-kystique, sein, Carcinoma, papillary, intracystic, breast

## Abstract

Le Carcinome papillaire intra-kystique du sein est une entité très rare et représente 0,5 à 1% de l'ensemble des carcinomes mammaires. Il se caractérise généralement par une croissance lente avec un bon pronostic. Nous rapportons 03 nouveaux cas prouvés histologiquement, chez des patientes traitées par chirurgie radicale ou conservatrice, suivie d'une radiothérapie sur le sein en place et une hormonothérapie à base de tamoxifen chez les deux patientes avec des récepteurs hormonaux positifs. Les trois patientes sont en bon contrôle locorégionale avec des suivis post-thérapeutiques de 12 à 18 mois.

## Introduction

Le carcinome papillaire intrakystique (CPIK) est une variante très rare de carcinome intracanalaire, ne constituant que 0,5% à 1% de tous les cancers du sein. Les lésions papillaires bénignes et malignes du sein sont très difficiles à distinguer sur la cytologie d’ ou la nécessité d'une étude histologique complétée par l'immuno-histochimie [1, 2]. Le CPIK se caractérise généralement par une croissance lente avec un bon pronostic. A l'occasion de ces 03 observations, nous rappelons les aspects diagnostiques, thérapeutiques et évolutifs de cette tumeur rare.

## Patient et observation

**Figure 1:**
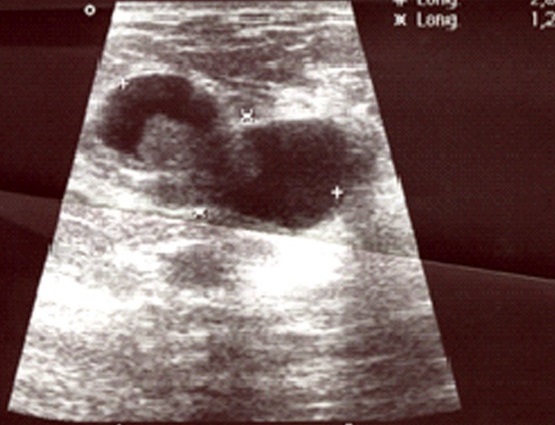
Image échographique montrant une masse du quadrant supero-externe du sein droit hétérogène à double composante kystique et tissulaire

**Figure 2:**
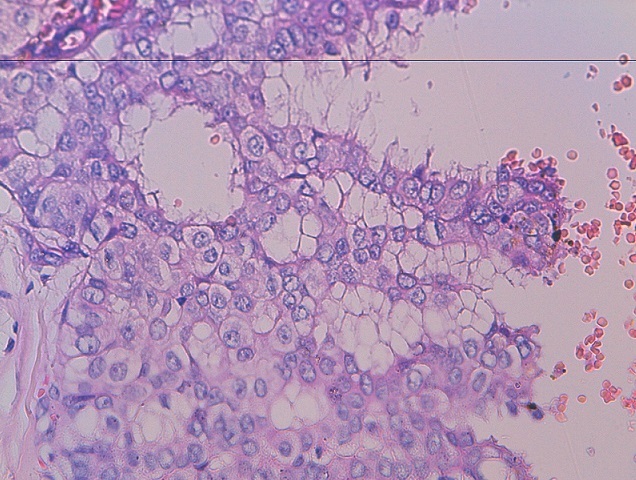
Hématoxyline-éosine (HE) x40: Histologie d'une biopsie d'un carcinome intra-kystique avec des excroissances papillaires, des noyaux atypiques, des chromatines agglutinées, et un pléomorphisme severe

**Cas 1:** Il s'agit d'une patiente de 44 ans, G2P2, encore réglée, sans antécédents pathologique particuliers qui a présenté 07 mois avant la consultation un nodule du sein droit, sans signes inflammatoires en regard ni écoulement mamelonnaire. A l'examen initial, le nodule siège au niveau du quadrant supero-externe du sein droit faisant 2×1,5 cm de diamètre, sans adénopathies axillaires. La mammographie couplée à l’échographie mammaire (Figure 1) parle d'une masse du quadrant supero-externe du sein droit à double composante kystique et tissulaire. Après tumorectomie large, l’étude anatomopathologique parle d'un aspect histologique d′un carcinome papillaire intra-kystique (Figure 2), avec des récepteurs hormonaux positifs et un Ki67 à 10%. La patiente a reçu une radiothérapie sur tout le sein droit à la dose de 50 Gy en 25 fractions de 2 Gy avec une bonne tolérance clinique puis mise sous tamoxifen 20 mg/j. elle est en bon contrôle locorégional et à distance avec un recul de 18 mois.

**Cas 2:** Il s'agit d'une patiente de 48 ans, G9P8, encore réglée, qui a consulté pour un nodule du sein gauche découvert à l'autopalpation, et évoluant depuis 6 mois. A l'examen clinique on note la présence d'un nodule du quadrant supéro-interne faisant 4 cm, ferme, mobile, sans signes inflammatoires en regard ni écoulement mamelonnaire. Les aires ganglionnaires axillaires étaient libres. La mammographie bilatérale a mis en évidence la présence de deux opacités à cheval des deux quadrants supérieures du sein gauche à contours flous. L’échographie mammaire révèle un fond de dystrophie micro kystique bilatérale avec présence au niveau du quadrant supéro-interne du sein gauche de deux lésions kystiques: l'une bi-lobulée renfermant un bourgeon charnu tissulaire hétérogène de 20 mm de grand axe. L'autre à paroi épaissie mesurant 14mm de grand axe. Une tumorectomie large est réalisée. L'examen histologique a montré des formations kystiques comblées par une prolifération d'allure carcinomateuse aux atypies modérées formées de papilles ou de structures glandulaires cribriformes. Cette prolifération tumorale intra-papillaire kystique évoque un carcinome intra-kystique. Les limites d'exérèse sont saines.les récepteurs hormonaux étaientt négatifs. La patiente a reçu une radiothérapie sur tout le sein gauche à la dose de 50 Gy en 25 fractions de 2 Gy avec une bonne tolérance clinique et un bon contrôle locorégional et à distance avec un recul de 14 mois.

**Figure 3:**
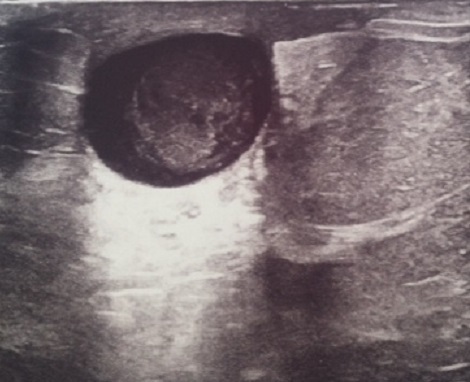
Image échographique montrant un kyste de 21 mm avec végétations intra-kystique

**Cas 3:** Il s'agit d'une patiente de 71 ans. G3 P2.ménopausée il ya plus de 15 ans. Diabétique sous metformine 850 2cp/j qui présentait depuis 18 ans un kyste retro-mamelonnaire traité par plusieurs ponctions itératives. La mammographie couplée à échographie mammaire bilatérales parlent d'un kyste de 21 mm avec végétations intra-kystique (Figure 3). La patiente a été opérée le 14/03/2013: L'extemporanée a répondu malin, complétée par une mastectomie. A l’étude histologique, il s'agit d'un carcinome papillaire intra-kystique, avec des récepteurs hormonaux positifs et un Ki67 à 10%. Elle est mise sous tamoxifene 20 mg/j. elle est en bon contrôle locorégionale et à distance avec un recul de 12 mois.

## Discussion

Le CPIK est une tumeur canalaire maligne rare, représentant 0,5 à 1% de l'ensemble des carcinomes mammaires. Il survient généralement après 40 ans avec un âge moyen qui varie de 55 à 67 ans selon les auteurs. Dans environ 50% des cas il est de siège central et plus précisément dans la région rétroaréolaire. La taille tumorale varie de 1 à 14 cm [1].

Le signe radiologique classique sur la mammographie est une opacité à contours nets, bien circonscrite, ovale ou polylobé. À l′échographie, il se présente sous forme d'une masse kystique complexe avec une composante solide montrant un flux vasculaire sur le doppler couleur. Ces caractéristiques radiologiques doivent faire suspecter cette forme rare de cancer du sein. L'imagerie par résonance magnétique du sein avec augmentation du contraste peut orienter le diagnostic en montrant le cloisonnement et les nodules muraux [2].

La biopsie de la lésion intéressant la portion solide est généralement plus informative. L’étude macroscopique retrouve au sein d'un kyste à paroi épaisse et fibreuse une formation polylobée, friable et hémorragique. En microscopie, l'architecture tumorale est papillaire le plus souvent avec des aspects cribriformes. Le diagnostic d'une invasion stromale est difficile [3].

La stratégie thérapeutiques est variables vu la rareté de cette forme de cancer du sein. La chirurgie mammaire conservatrice avec exérèse large est la plus utilisée, néanmoins, dans certains cas, la mastectomie avec ou sans reconstruction mammaire immédiate peut être proposée (par exemple, les grosses tumeurs, insuffisance des marges, la récidive et la préférence de la patiente). Les métastases ganglionnaires sont exceptionnelles. La chirurgie ganglionnaire axillaire sous forme de biopsie du ganglion sentinelle ou du curage axillaire est à éviter pour épargner aux patientes la morbidité de curage axillaire [4]. La recherche des arguments pour soutenir la théorie que la radiothérapie adjuvante réduit considérablement le risque de rechute locale chez les patientes qui ont eu une chirurgie conservatrice du sein en cas des CPIK est toujours en cours. Cependant, de nombreux articles et données publiées recommandent la radiothérapie chez les jeunes femmes de moins de 50 ans, dans les formes associées à l′invasion et ou à un carcinome canalaire in situ(CCIS) [5]. Le faible potentiel métastatique et d′invasion vasculaire rend la chimiothérapie non obligatoire. L'hormonothérapie adjuvante principalement avec le tamoxifène devrait être prescrite pour réduire le risque de récidive locale en cas de récepteurs hormonaux positifs. En dépit de ces principes généraux, le traitement optimal du CPIK reste controversé [6].

Le CPIK est caractérisé généralement par une croissance lente avec un très bon pronostic comparativement aux autres carcinomes intra-canalaires. Lefkowitz rapporte un taux de survie à 10 ans sans maladie de 91% [7].

## Conclusion

Le carcinome mammaire intra-kystique représente une entité particulière des cancers du sein par sa survenu à un âge avancé, sa croissance intra-canalaire lente et son architecture papillaire. L’échographie est le maitre examen diagnostique. Le diagnostic est confirmé par une microbiopsie de la portion charnue. La base du traitement est la mastectomie ou une tumorectomie conservatrice du sein. La radiothérapie et / ou une hormonothérapie adjuvantes sont à considérer dans les cas appropriés.
